# Genome Sequence of a Novel Canine Picornavirus Isolated from an American Foxhound

**DOI:** 10.1128/genomeA.00338-17

**Published:** 2017-05-18

**Authors:** Erica E. Norby, Richard G. Jarman, Paul B. Keiser, Leonard N. Binn, Jun Hang

**Affiliations:** Walter Reed Army Institute of Research, Silver Spring, Maryland, USA

## Abstract

A candidate new canine picornavirus was isolated from a respiratory swab collected from an American foxhound (*Canis lupus familiaris*) in 1968. The assembled genome sequence of strain A128thr is 7,618 bases in length, comprising a complete protein-coding sequence of the 2,213-amino-acid polyprotein and partial terminal untranslated sequences.

## GENOME ANNOUNCEMENT

Canine respiratory viruses cause asymptomatic to severe diseases in dogs and other hosts (1, 2). Contagious respiratory viral infection in procured dogs was a significant problem in the preparation of these animals for laboratory studies or military service (3, 4). During the 1960s and 1970s, we made considerable efforts to isolate potentially causative viruses (5, 6). The isolates were thoroughly examined to characterize the chemical and physical properties, as well as their serological identities, but molecular or genomic investigations had not been conducted until recently. The acquired genome sequence indicated that the isolated virus A128thr represents a new canine picornavirus species.

The virus A128thr was recovered in 1968 from the throat swab of an asymptomatic foxhound (*Canis lupus familiaris*) from Florida and categorized as a picorna-like virus based on its virological characteristics. It produced cytopathic effects (CPE) in viral cultures using primary dog kidney (PDK) cells, canine cell lines, and also in several other mammalian cells, including human diploid fibroblast cells (WI-38). The virus was found to be nonenveloped, be approximately 30 μm in diameter, and to contain an RNA genome. Nucleic acids of A128thr were extracted from the supernatant of PDK cell culture (five passages) using the QIAamp viral RNA purification kit (Qiagen Sciences, Germantown, MD) and subjected to random reverse transcription, random PCR amplification, and next-generation sequencing using a MiSeq sequencer and reagents (Illumina, Inc., San Diego, CA) (7, 8). Sequence data were assembled using Roche GS analysis software version 2.9 (Roche 454 Life Sciences, Branford, CT) and analyzed with BLAST programs (http://blast.ncbi.nlm.nih.gov/Blast.cgi) and the Geneious 8.1.7 software (Biomatters, Auckland, New Zealand).

The assembled genome sequence is 7,618 bases in length, comprising a complete protein-coding sequence and partial terminal untranslated sequence (UTR). In total, 404,327 MiSeq reads and 91,393,658 bases of sequence data were mapped to this genome sequence, with an average sequence alignment depth of 12,002-fold. The genome contains a complete open reading frame of 2,213 amino acids (accession no. APY24210). The amino acid sequence shared 44.2% or lower identity with a polyprotein of *Miniopterus schreibersii* picornavirus 1 (accession no. AFK85007) and those of other bat picornaviruses (9, 10). The amino acid residue similarities between A128thr and *Miniopterus schreibersii* picornavirus 1 are 63.3% (1,415/2,236 amino acids) for the polyprotein and 60.2% (486/807 amino acids), 62.9% (342/544 amino acids), and 69.9% (573/820 amino acids) for regions P1 (capsid proteins VP4 to VP1), P2 (nonstructural proteins 2A-2C), and P3 (proteins 3A, 3B, 3C proteinase, and 3D RNA-dependent RNA polymerase), respectively. Interestingly, A128thr exhibits only about 23% amino acid identities and 41 to 52% similarity in various regions with canine picornavirus strains 6D, 244F, and 325F (11, 12). The novelty of the virus was shown by its low sequence similarity for the gene products with known canine picornaviruses and with other picornaviruses. We provisionally designate the virus canine picornavirus isolate A128thr, because it was isolated from a dog and is capable of infecting canine primary cells and cell lines. It is tentatively classified in the family *Picornaviridae*, with its genus nomenclature yet to be clarified (12, 13). Further studies are needed to elucidate its host spectrum, tissue specificity, infectivity, and pathogenicity to human and animals.

### Accession number(s).

The genome sequence for canine picornavirus isolate A128thr was deposited in GenBank under accession no. KY512802.
